# Sequence similarity searches for morphine biosynthesis enzymes in bacteria yield putative targets for understanding associations between infection and opiate administration

**DOI:** 10.1099/jmm.0.001001

**Published:** 2019-05-20

**Authors:** Shing Hei Zhan, Leon French

**Affiliations:** 1 Department of Zoology, University of British Columbia, Vancouver, Canada; 2 Krembil Centre for Neuroinformatics, Centre for Addiction and Mental Health, Toronto, ON, Canada; 3 Campbell Family Mental Health Research Institute, Centre for Addiction and Mental Health, Toronto, ON, Canada; 4 Division of Brain and Therapeutics, Department of Psychiatry, University of Toronto, Toronto, ON, Canada; 5 Institute for Medical Science, University of Toronto, Toronto, ON, Canada

**Keywords:** morphine, ESKAPE, *Pseudomonas aeruginosa*, bacteria, opiates

## Abstract

Exploiting the immunosuppressive, analgesic and highly addictive properties of morphine could increase the success of a bacterial pathogen. Therefore, we performed sequence similarity searches for two morphine biosynthesis demethylases in bacteria. For thebaine 6-O-demethylase and codeine O-demethylase, we found strong alignments to three (* Pseudomonas aeruginosa , Klebsiella pneumoniae* and *Acinetobacter baumannii *) of the six ESKAPE pathogens (* Enterococcus faecalis *, *Staphylococcus aureus *, *K. pneumoniae *, *A. baumannii *, *P. aeruginosa* and *Enterobacter* species) that are commonly associated with drug resistance and nosocomial infections. Expression of the aligned sequence found in *P. aeruginosa* (NP_252880.1/PA4191) is upregulated in isolates obtained from cystic fibrosis patients. Our findings provide putative mechanistic targets for understanding the role of morphine in pathogenicity.

## Introduction

Several parasites are known to cause behavioural changes in their hosts. Studies on the molecular mechanisms that are altered by these parasites are lacking. A bioinformatic analysis discovered two genes that encode tyrosine hydroxylase in the genome of the *Toxoplasma gondii* parasite. These enzymes were found to have substrate specificity [1]. Tyrosine hydroxylase is the rate-limiting enzyme in dopamine synthesis, linking reward-motivated behaviour to infection. This finding motivated a follow-up study that found that dopaminergic cells infected with *T. gondii* had enhanced dopamine release [2]. Beyond the devastating effects on the developing nervous system, *T. gondii* infection is also associated with schizophrenia [3, 4], which has long been associated with dopamine, including a genome-wide significant association in the dopamine receptor 2 gene [5]. Such findings, which began with bioinformatics analyses, provide target mechanisms for understanding how parasites alter host behaviour.

Inspired by the above findings, we undertook protein sequence searches for enzymes of interest. We focused on morphine synthesis due to the immunosuppressive, analgesic and highly addictive properties of the compound [6]. Sequences encoding two key enzymes for morphine synthesis in the opium poppy (*Papaver somniferum*) have been identified [thebaine 6-O-demethylase (T6ODM) and codeine O-demethylase (CODM)] [7]. Previously, a parasitic worm that infects pigs (*Ascaris suum*) was shown to synthesize morphine while lacking expression of an opioid receptor [8]. Further, the expression of a morphine-like compound and a μ opiate receptor was detected in *Dicrocoelium dendriticum*, a parasite fluke [9]. These findings suggest that these parasites may use morphine to evade the host immune system and possibly induce analgesia.

Beyond eukaryotic parasites, we surmised that bacteria may encode enzymes involved in morphine synthesis. Vouga and Greub have described how the emergence and reemergence of infectious diseases caused by bacterial pathogens present a major public health problem [10]. As a result, they called for increased resources for research aimed at understanding bacterial pathogenicity. Such efforts are supported by the availability of thousands of genomes that catalogue the wide diversity of bacterial pathogens [11, 12]. Across the protein sequences translated from these genomes, we searched for the sequences of two enzymes involved in morphine biosynthesis to understand the associations between infection and opiates.

## Methods

### Sequence search and analysis

We performed protein sequence similarity searches with the Basic Local Alignment Search Tool from the National Center for Biotechnology Information (NCBI-blast) [13]. Specifically, protein–protein blast (blastp) was used with the default settings and with the organism restricted to bacteria (taxid: 2) in the non-redundant protein database (nr). To limit our analysis, we focused on the top 100 blast hits for T6ODM and CODM. To further examine the sequence similarity of T6ODM, CODM and their bacterial homologues, we built a multiple sequence alignment (MSA) with Multiple Sequence Comparison by Log-Expectation (muscle) version 3.8.31 using the default settings [14]. We added functional domain annotations to the MSA. These annotations were provided by UniProtKB for T6ODM and CODM [15]. We checked whether homologues of the same functional domains are present in the bacterial homologues of T6ODM and CODM using InterProScan version 5.34–73.0 via its web service [16]. JalView version 2.10.5 was used to visualize the alignment [17].

### Gene expression analysis

Microarray expression data from a study of expression data from GDS2869 was downloaded from the Gene Expression Omnibus (GEO) database. This study assayed expression of *Pseudomonas aeruginosa* isolates from the lungs of cystic fibrosis patients [18]. Differential expression was tested with a Mann–Whitney U test between the percentile rank expression from the reference strain (PAO1) and specimens obtained from cystic fibrosis patients (*in vivo* and minimal media isolates).

## Results

**Fig. 1.**
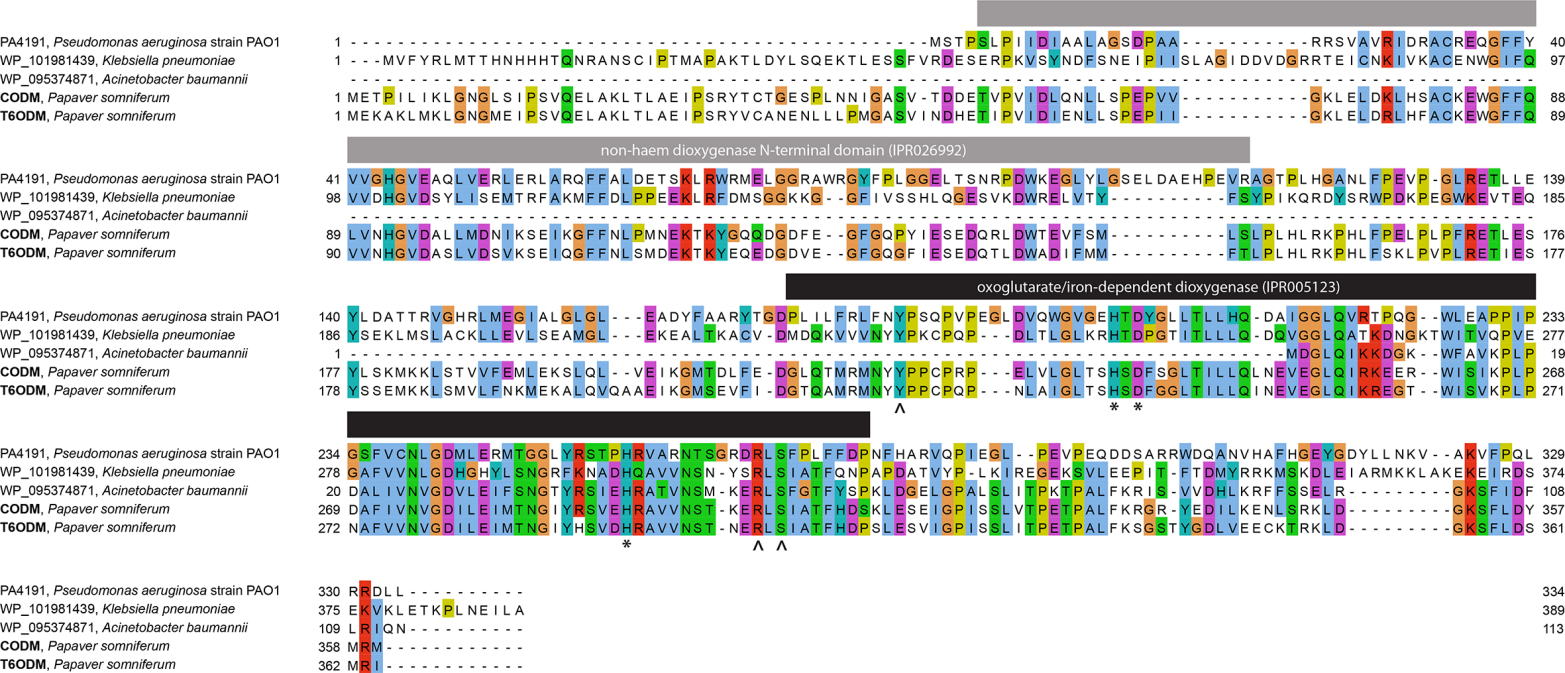
Visualization of the multiple alignment of the selected sequences. The two functional domains (non-haem dioxygenase N-terminal domain and oxoglutarate/iron-dependent dioxygenase) in T6ODM and CODM identified by UniProtKB and InterProScan are represented by grey and black bars, respectively. Additionally, the 2-oxoglutarate- and iron cation-binding sites of the oxoglutarate/iron-dependent dioxygenase domain in T6ODM and CODM are marked with ^ and * characters, respectively (from UniProtKB). Conserved amino acids (dynamically determined by JalView) are highlighted with the clustal colour scheme: hydrophobic (blue); negatively charged (magenta); positive (red); polar (green); prolines (yellow); cysteines (pink); glycines (orange); and aromatic (cyan).

blastp searches restricted to bacteria for T6ODM and CODM returned numerous hits, with the top alignments having expected values of 3.9×10^−31^ or lower. Motivated by the discovery of a dual-purpose enzyme in *T. gondii*, we restricted our analysis to the 70 proteins that were found in the top 100 hits from both the T6ODM and CODM searches (Table S1, available in the online version of this article). This high overlap was not unexpected, given that the pairwise identity between the protein sequences of T6ODM and CODM is 73 %. We note the presence of 3 opportunistic pathogens, *P. aeruginosa *, *Klebsiella pneumoniae* and *Acinetobacter baumannii *, within the 17 organisms listed in the top 70 shared hits (Table 1). These three make up half of the ESKAPE acronym (comprising *Enterococcus faecalis *, *Staphylococcus aureus *, *K. pneumoniae *, *A. baumannii *, *P. aeruginosa* and *Enterobacter* species) that combines species that are commonly associated with drug resistance and nosocomial infections [19]. We did not find strong alignments for the remaining three species, but note that WP_129362402.1 from *Enterobacter* species (identity <29.5 %, E value >10^−25^ and rank >1080 for both T6ODM and CODM alignments) ranked highest for clinical importance. The matching sequences from the selected pathogens were aligned to T6ODM and CODM (Fig. 1).

**Table 1.** Top-ranked alignments from the ESKAPE bacterial pathogens that appear in the top 100 hits from both the T6ODM and CODM searches. The rank column provides the ranking within these separate searches. The full listing is available in Table S1

		Thebaine 6-O-demethylase (T6ODM)			Codeine O-demethylase (CODM)		
Identifier	Description and organism	Identity	E value	Rank	Identity	E value	Rank
WP_101981439	Hypothetical protein (* Klebsiella pneumoniae *)	32.99	8.33E–48	4	33.444	8.46E–47	5
WP_095374871	Hypothetical protein (* Acinetobacter baumannii *)	55.357	3.57E–34	10	60.36	6.53E–40	7
WP_065285055	Isopenicillin N synthase family oxygenase (* Pseudomonas aeruginosa *)	31.65	1.41E–32	20	32.203	9.77E–36	20

T6ODM, CODM and their bacterial homologues contain an oxoglutarate/iron-dependent dioxygenase functional domain (IPR005123, from UniProtKB and InterProScan). Further, the non-haem dioxygenase N-terminal domain (IPR026992) is annotated in all of the sequences except the shorter *A. baumannii* sequence. Moreover, T6ODM (UniProtKB ID: D4N500) and CODM (D4N502) are annotated with three 2-oxoglutarate-binding sites and three iron cation (the enzymes’ cofactor)-binding sites, all of which are situated in the dioxygenase domain. All six binding sites appear to be highly conserved in the aligned sequences (Fig. 1)**,** suggesting that the bacterial homologues are capable of similar enzymatic functions to T6ODM and CODM.

**Fig. 2.**
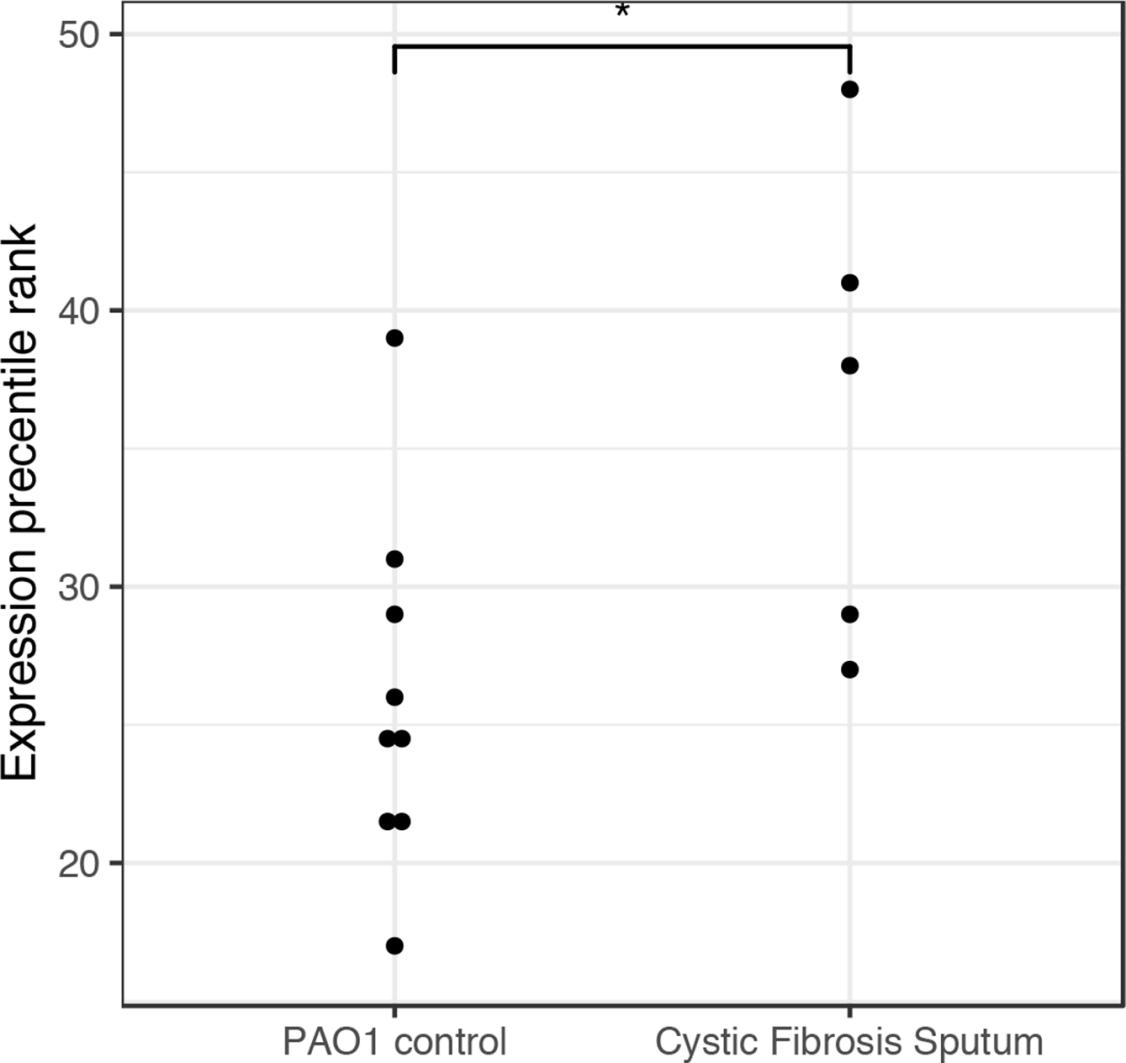
Dot plot of expression of PA4191 in GDS2869 (*P<0.05, two-sided Mann–Whitney U test).

*P. aeruginosa* is the species listed for 40 of the 70 hits, which may be due to this human pathogen being overrepresented in sequence databases. Sequences from *Pseudomonas denitrificans* and HMSC05H02 species were also found within the top 70. To focus our analyses, we examined the top hit (WP_023129413), which maps to the NP_252880.1 protein and the PA4191 locus tag in the *P. aeruginosa* reference genome of the PAO1 laboratory strain. The NP_252880.1 protein sequence is 334 amino acids in length, with 297 aligning to T6ODM (31.3 % identity) and 295 aligning to CODM (32.2 % identity). In the Pseudomonas Genome Database, PA4191 is labelled as a probable iron/ascorbate oxidoreductase with a cytoplasmic subcellular localization [20]. Manual inspection of the 23 experiments that assayed PA4191 gene expression revealed a clinically relevant study with a significant upregulation of expression in specimens from cystic fibrosis patients (*P*<0.05, uncorrected for the 23 reviewed experiments, Fig. 2).

Using ortholugeDB [21], we downloaded the 112 orthologue clusters for PA4191. Within these orthologues, *Yersinia pestis *, which causes the plague, was the most frequent species, with 12 strains, followed by *Campylobacter jejuni* (9) and *Yersinia pseudotuberculosis* (4).

Three sequences from *A. baumannii* that were in the 100 hits for both T6ODM and CODM were short. Two were partial coding sequences (PAL70652 and PAL63834), while a third was listed as a hypothetical protein (WP_095374871, 113 aa). The sequence from *K. pneumoniae* that aligned to both T6ODM and CODM is listed as a hypothetical protein (WP_101981439.1). This sequence is 389 amino acids in length, with 291 aligning to T6ODM (33 % identity) and 302 aligning to CODM (33 % identity). Across all bacteria, this alignment is ranked within the top five based on expected values for both T6ODM and CODM. We did not find any orthologue or expression data for the *A. baumannii* or *K. pneumoniae* proteins using the ortholugeDB and GEO databases.

## Discussion

We performed a protein sequence search that revealed unexpected sequence similarities between two proteins involved in morphine biosynthesis and three human pathogens. These three make up half of the ESKAPE list of antibiotic-resistant and nosocomial pathogens. Morphine has been shown to enhance pathogenicity for two of the three pathogens. Specifically, morphine administration has been found to activate *P. aeruginosa* virulence expression to cause lethal sepsis in mice [22]. In the same study, morphine was also shown to be a chemoattractant for *P. aeruginosa *. Beyond morphine, *P. aeruginosa* is sensitive to other opioids [23, 24]. For *A. baumannii *, morphine treatment increased mortality in mice infected with the pathogen [25]. This synergistic effect was blocked by an opiate antagonist (naltrexone). We note that *E. faecalis *, an ESKAPE pathogen that was not found in our search, has been shown to demonstrate increased adhesiveness upon morphine exposure in rats [26]. Unlike in the *A. baumannii* and *P. aeruginosa* studies, an increase in mortality was not demonstrated. Overall, these rodent studies suggest that morphine is a key molecule for these opportunistic pathogens. These animal studies are supported by findings of increased endogenous morphine levels in patients with sepsis [27]. Based on our findings of sequence similarities, we speculate that morphine may be synthesized by pathogens as a mechanism to increase virulence.

Another species of the *Pseudomonas* genus, *P. putida *, has been shown to convert morphine into several other opioids with improved analgesic qualities (hydromorphone and dihydromorphine) [28]. Recently, the complete biosynthesis of morphine was engineered in yeast [29]. This opioid biosynthesis strain included genes from *P. putida *, but it also relied on T6ODM from *Papaver somniferum*. T6ODM and CODM have strong sequence alignments in *P. putida *, but they did not make into the top 100 alignment lists (percentage identity <27 % and E values >10^−30^). While *P. putida* has previously been associated with opioid biosynthesis, *P. aeruginosa* contains genes with higher sequence similarities to the poppy enzymes.

### Limitations and future work

We found increased PA4191 expression in specimens from cystic fibrosis patients. The high incidence of *P. aeruginosa* infections in cystic fibrosis patients and the availability of opioid antagonists suggest that these preliminary findings should be followed up.

*P. aeruginosa* is known to have a large virulence gene repertoire, which allows the pathogen to infect a broad range of plant and animal hosts [30, 31]. Relative to the many other virulence factors, PA4191 may not play an essential role in *P. aeruginosa* infections. However, the immunosuppressive, analgesic and highly addictive properties of morphine would be useful to *P. aeruginosa* and possibly other human pathogens.

The finding of tyrosine hydroxylase in *T. gondii* that inspired this work provides a guide to follow-up studies. Gaskell *et al*. tested the substrate specificity of the enzymes for phenylalanine and tyrosine [1]. They also demonstrated the bifunctional nature of the enzyme, which we sought by intersecting blast results for T6ODM and CODM. In a follow-up study, the same group found that infected cells had increased dopamine metabolism [2]. Similar experiments could be performed for the genes found in *A. baumannii *, *P. aeruginosa* and *K. pneumoniae* to assess the activity and function of the encoded proteins. Specifically, changes to pathogenic virulence after the deletion, overexpression, or mutation of these genes could be experimentally tested. The biosynthetic pathway for endogenous morphine has been detailed in human cells, providing substrates for pathogenic enzymes that synthesize morphine [32]. While we identified several interesting sequences, we stress that sequence similarity does not ensure functional similarity. We note that similar sequences are also found in non-pathogenic bacteria. Additional study is required to determine whether the aligned genes can synthesize or convert morphine, and can increase virulence or affect host behaviour.

## Supplementary Data

Supplementary material 1
